# State of the Art of BIM Integration with Sensing Technologies in Construction Progress Monitoring

**DOI:** 10.3390/s22093497

**Published:** 2022-05-04

**Authors:** Ahmed R. ElQasaby, Fahad K. Alqahtani, Mohammed Alheyf

**Affiliations:** 1 Department of Civil Engineering, College of Engineering, King Saud University, P.O. Box 800, Riyadh 11421, Saudi Arabia; alheyf@ksu.edu.sa; 2 Department of Civil Engineering, College of Engineering, Portsaid University, P.O. Box 42526, Portsaid 42511, Egypt

**Keywords:** sensing technologies, automated progress monitoring, object recognition, meta-analysis

## Abstract

The necessity for automatic monitoring tools led to using 3D sensing technologies to collect accurate and precise data onsite to create an as-built model. This as-built model can be integrated with a BIM-based planned model to check the project’s status based on algorithms. This article investigates the construction progress monitoring (CPM) domain, including knowledge gaps and future research direction. Synthesis literature was conducted on 3D sensing technologies in CPM depending on crucial factors, including the scanning environment, assessment level, and object recognition indicators’ performance. The scanning environment is important to determine the volume of data acquired and the applications conducted in the environment. The level of assessment between as-planned and as-built models is another crucial factor that could precisely help define the knowledge gaps in this domain. The performance of object recognition indicators is an essential factor in determining the quality of studies. Qualitative and statistical analyses for the latest studies are then conducted. The qualitative analysis showed a shortage of articles performed on 5D assessment. Then, statistical analysis is conducted using a meta-analytic regression model to determine the development of the performance of object recognition indicators. The meta-analytic model presented a good sign that the performance of those indicators is effective where [*p*-value is = 0.0003 < 0.05]. The study is also envisaged to evaluate the collected studies in prioritizing future works from the limitations within these studies. Finally, this is the first study to address ranking studies of 3D sensing technologies in the CPM domain integrated with BIM.

## 1. Introduction

Remote sensing has been defined as one of the broadest areas of science because of its various applications in different fields such as geography, medicine, and engineering. Researchers labeled remote sensing an inventive science to remotely know information about an object [1]. In contrast, another study described remote sensing as a “renaissance at a distance.” [2]. Remote sensing aims to extract remotely sensed images using four crucial correlated processes. At first, the physical objects involve live beings such as humans or animals and inanimate beings such as buildings, land, and water. Second, the sensor data is shaped by recording the electromagnetic radiation emitted or reflected from the examined object. Moreover, the extracted information analyzes captured sensor data to solve practical problems. Finally, applications are the last element encompassing many aspects of science, such as geology, geography, engineering, and medicine [3].

In recent years, remote sensing technologies have played a crucial role in developing the architectural, engineering, and construction (AEC) industry. These technologies included global positioning systems (GPS), radio frequency identification (RFID), ultra-wideband (UWB) tracking system, image-based processing, and laser scanners (LS).

Several studies have been made to discuss 3D sensing technologies in construction. At first, researchers developed techniques to change manual inspection to automated inspection to increase the response time to any delays [4,5]. Then, studies began to suggest remote sensing technologies in surveying activities. Afterward, restoring historic buildings was another area for 3D sensing technologies. This area introduced the relationship between 3D sensing technologies and a new paradigm called building information modeling (BIM). On one hand, BIM accurately assembles “as planned” models into computer-generating programs to develop a spatial representation of objects [6,7]. 3D BIM-based model is formed by linking the project’s information with 3D model. On the other hand, remote sensing technologies could review the status of the building by assembling “as-built” models. As a result, a developed model was created by integrating those technologies to restore, record, and improve historic buildings [8,9].

Then, researchers turned to integrating BIM and remote sensing technologies to monitor the progress of activities in real-time to reduce schedule and cost overrun [10]. The reason behind that is the usage of BIM through dimensions. For example, a 4D BIM-based model, which is also referred to as the schedule model. The scheduling dimension has been specifically designated to establish the activities’ sequence over time. A cost model is another BIM dimension known as a 5D BIM-based model. The cost dimension is to track the costs of activities over time.

Moving forward to the latest review articles, they explored different insights to find knowledge gaps and recommend future directions in the construction industry. For example, Patel pointed out some visions into the CPM domain using scientometric analysis to draw a broad picture of CPM [11]. Another article focused on the BIM research domain and its development from data collection to information integration and knowledge management [12]. It also used science mapping-based analysis to draw theoretical and practical references for future research on BIM. However, other researchers reviewed articles on machine learning methods for point cloud processing in construction and infrastructure applications [13]. Another researcher pointed out 3D point cloud data for different construction purposes such as 3D model reconstruction, geometry quality inspection, and other applications [14].

In contrast, few studies focused on monitoring the automation of indoor progress by providing a systematic literature survey [15]. However, previous studies intended to pave the way for advancing different domains in the construction industry. A few topics in the construction industry still need addressing. One of these topics is surveying a correlation between certain data acquisition technologies and certain environments. Another topic is to address the gaps in the BIM integrated with the CPM applications. Another topic is surveying the performance of object detection algorithms to determine the development of these algorithms over the years and quantify the quality of studies.

There are two main objectives of this research. Firstly, to present a literature synthesis and investigate the current state of research on integrated BIM with 3D sensing technologies in CPM for knowledge gaps and future works. This research was assessed according to the scanning environment, assessment levels, and performance of object recognition indicators. Secondly, to investigate the efficacy of object recognition indicators’ performance using meta-analysis.

Today, such a view is necessary as there is a lack of review of integration between BIM and sensing technologies in construction progress monitoring. Accordingly, the findings of this research are expected to partake in the current state of research in the CPM. This research also highlights the strengths and weaknesses of studies related to BIM and 3D sensing technologies.

This paper focused on studies between 2007 and 2021. The reason behind that was researchers mainly used a traditional CPM before 2007. The traditional CPM mostly depends on daily or weekly reports from the site collected manually and uploaded to a computer after analysis of these reports. After that, Patel pointed out a huge transformation of research toward automation and visualization in the CPM field [11]. The article proceeds by conducting a qualitative and statistical analysis of previous studies. This qualitative analysis was assessed according to the scanning environment, assessment level, and performance of object recognition indicators.

Those criteria were chosen for particular reasons. For example, the scanning environment criterion was preferred to investigate how frequently 3D sensing technologies are used in different environments and whether there is a correlation between certain data acquisition technologies and different environment sets or not. Another example, the level of assessment criterion, was selected to investigate the development of CPM-integrated applications between as-built and BIM-based models. In comparison, the performance of object recognition indicators criterion was preferred to examine the quality of studies based on object recognition algorithms’ development.

For further investigation of the performance of object recognition indicators, the article conducted a statistical analysis of previous studies using meta-analysis(for example, the steps to conduct a meta-analysis and how they are utilized to analyze the literature findings). After that, the article results are demonstrated. Finally, the paper concludes with an overall summary of the results.

## 2. Research Outlines

This research includes the latest studies published in this area over the past decades. The literature search focused on highly regarded journals in civil engineering informatics, construction engineering and management, remote sensing, applied science, and automation in construction. Papers published in or after 2007 are only involved as before 2007, papers were used traditional approaches. A total of 46 articles were collected using a literature search in Google Scholar and the digital library of King Saud University using keyword search. The keyword used for the literature search is a combination of data acquisition technologies and integration with BIM in construction progress monitoring. Keywords representing data acquisition technologies include “RFID,” UWB,” GPS,” Image Processing,” Laser Scanner” (See Section 3). Keywords representing areas include “integrated with BIM,” monitoring and control, “construction progress monitoring,” Progress tracking.” Selected papers must use point cloud data integrated with BIM for progress monitoring purposes. However, these previous procedures are detailed in extracting articles.

**Figure 1:**
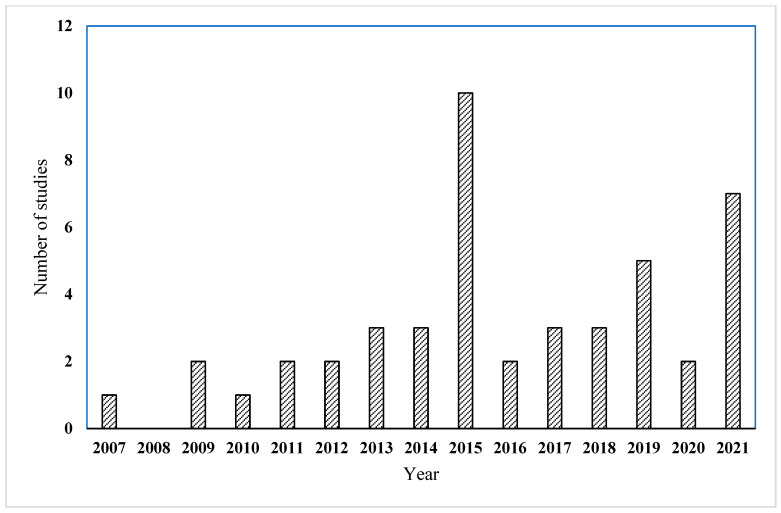
Number of articles per year regarding 3D sensing technologies integrated with BIM in CPM.

Based on the previous literature search, a total of 46 research papers were reviewed between 2007 and 2021 due to the traditional way to monitor progress before 2007. Figure 1 shows the number of articles per year between 2007 and 2021. Figure 1 shows a continuous increase in the last ten years. In the last five years, more than 20 papers have been found, indicating the importance of this research topic.

A critical analysis is then developed to discuss the collected studies according to the following criteria:

1. Scanning environment: the environment where 3D sensing technology captures the necessary as-built data (indoors, outdoors, or both)
2. Level of assessment: The level of progress monitoring data between the as-built model and as-planned model [three-dimensional (3D), four-dimensional (4D), or five-dimensional (5D)]
3. Performance of object recognition indicators [recall, accuracy, and precision] (see Section 3)

**Figure 2:**
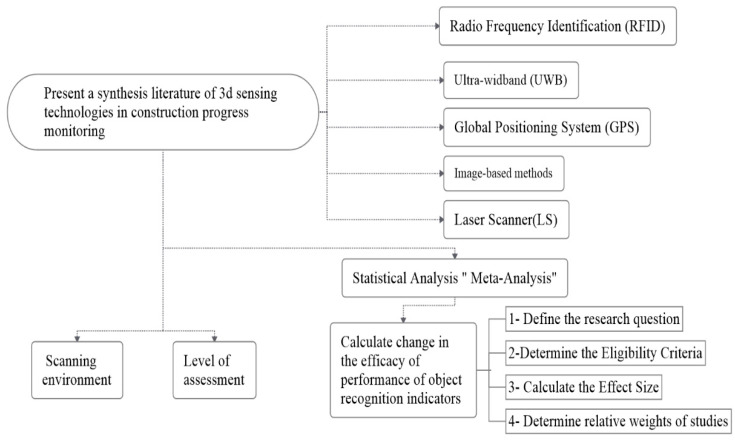
Research Flowchart.

Furthermore, statistical analysis is discussed to evaluate the efficacy of object recognition indicators’ performance using meta-analysis. The research flowchart is shown in Figure 2. Further explanation of the methodological steps is provided hereunder.

## 3. Overview on 3D Sensing Technologies in Construction

Nowadays, construction projects have many issues. The massive amount of data and the lack of cooperation between construction departments are two core problems. As a result, construction firms turned their attention to multidimensional planned models such as BIM models. For onsite monitoring and control purposes, 3D sensing technologies also helped form 3D as-built models. As mentioned before, these technologies were Laser scanners (LS), global positioning systems (GPS), radio frequency identification (RFID), ultra-wideband (UWB) tracking systems, and image-based processing [16]. The previous research urged that laser scanning and image-based processing are the most used 3D sensing technologies in the construction phase. However, the laser scanner is accurate in obtaining 3D onsite data. It is expensive and needs experienced operators [10].

Furthermore, image-based methods can generate 3D or 4D models. Image processing abilities can produce models based on geometrical information. However, like laser scanning, image-based methods have limitations, such as being time-consuming as it needs more overlapping images in various places in the project area [17]. 

Moreover, unmanned aerial vehicle (UAV) is another image-based method. A UAV is an aircraft that flies either autonomously or with remote control. UAV can cover the investigation area and obtain various data types such as videos or images [18].

The quality of 3D sensing technologies is crucial for developing as-built models [19]. In other words, a classification of predicted and correctly sensed point clouds should be explained. Therefore, a confusion matrix is used to summarize point clouds’ performances through different conditions, as described in Table 1. A confusion matrix is mainly used in machine learning to summarize predictive results on a classification problem. It also visualizes the algorithm’s performance. It usually contains two rows and two columns that report the number of should be (true positives, true negatives, false negatives, and false positives).

These conditions revealed some common performance indicators. Those indicators involved recall, accuracy, and precision [20,21]. Firstly, recall rate is the percentage of correctly sensed model objects present in the scans. Secondly, the accuracy (specificity) rate is the percentage of all sensed model objects in all observation cases. Finally, the latest indicator is the precision rate, and it is defined as the percentages of correctly sensed model objects that are actually in the scan [20]. These benchmarks are interpreted below in Equations (1)–(3), which would help indicate the performance level of object recognition.

(1) $$\mathrm{Recall}\;=\;\frac{\mathrm{True}\;\mathrm{Positive}\;\left(\mathrm{TP}\right)}{\mathrm{True}\;\mathrm{Positive}\;\left(\mathrm{TP}\right)\;+\;\mathrm{False}\;\mathrm{Negative}\;\left(\mathrm{FN}\right)}$$

(2) $$\begin{array}{l} \mathrm{Accuracy}\;=\;\\ \frac{\mathrm{True}\;\mathrm{Positive}\;\left(\mathrm{TP}\right)\;+\;\mathrm{True}\;\mathrm{Negative}\;(\mathrm{TN})}{\mathrm{True}\;\mathrm{Positive}\;\left(\mathrm{TP}\right)\;+\;\mathrm{True}\;\mathrm{Negative}\;\left(\mathrm{TN}\right)\;+\;\mathrm{False}\;\mathrm{Positive}\;\left(\mathrm{FP}\right)\;+\;\mathrm{False}\;\mathrm{Negative}\;(\mathrm{FN})} \end{array}$$

(3) $$\mathrm{Precision}\;=\;\frac{\mathrm{True}\;\mathrm{Positive}\;(\mathrm{TP})}{\mathrm{True}\;\mathrm{Positive}\;\left(\mathrm{TP}\right)\;+\;\mathrm{False}\;\mathrm{Positive}\;(\mathrm{FP})}$$

### 3.1. Radio Frequency Identification (RFID)

Radio frequency identification (RFID) is one of the first used 3D sensing technology in the AEC industry. An RFID system mainly consists of readers, antennas, and tags. Tags are installed on the assets that need to be tracked. Reading data from tags is the antennas’ job. Readers transmit collected data for further processing and analysis into host computers. Readers also are typically pinpointed around the search area [22,23,24]. RFID-based systems mainly target tracking and location information for assets construction. RFID-based systems could also integrate with the multidimensional BIM technique to auto-update the progress of construction activities in real-time [22,23,24,25,26,27].

Table 2 depicts the studies conducted by RFID systems in the CPM field between 2007 and 2021. Very few studies were performed using 4D assessment [22,27]. In contrast, the remaining studies were performed using 3D assessment. While to the best of the authors’ knowledge, no studies were conducted using 5D assessment.

While regarding the number of 3D sensing technology used, [23] was conducted using an RFID-based system and a laser scanner. However, few studies were performed indoors and outdoors [23], while the rest were conducted indoors. Amongst studies that used RFID systems, very few studies revealed object recognition indicators (i.e., recall, accuracy, and precision measures) [26].

### 3.2. Ultra-Wideband (UWB)

Ultra-wideband (UWB) is one of the most promising positioning 3D sensing technologies. UWB-based systems typically consist of tags and sensors. UWB signals are emitted from tags and received by sensors around the sensing area. The location of objects is tracked using both the arrival time difference between different sensors and the angle of arrival at each sensor. A UWB-based system can track resources accurately and improve workplace safety [28]. Integrating BIM with UWB-based systems would result in a better information flow between the two systems and auto-monitor and auto-report work progress [29,30,31].

Table 3 depicts the studies conducted by UWB systems in the CPM field between 2007 and 2021. All studies were performed using a 3D assessment. While to the best of the authors’ knowledge, no studies were conducted using 4D or 5D assessment. While regarding the number of 3D sensing technology used, the data acquisition pointed by shahi was performed using a fusion of UWB and LS-based methods. Nevertheless, some studies were conducted indoors [31], while very few were implemented indoors and outdoors [29]. In contrast, one study was performed outdoors [30].

Amongst these studies, minimal studies used indicators of object recognition performance (i.e., accuracy measure) [31]. The study findings revealed that the accuracy results obtained using a Light Emitting Device (LED) indicator was higher than those without an LED.

### 3.3. Global Positioning System (GPS)

Global positioning system (GPS) is one of the most used 3D sensing technologies for tracking and pinpointing resources in the AEC industry. It also can retrieve positioning data from components in different scanning environments [32]. For example, GPS can track any resource in construction sites with only a GPS device placed on it and obtain real-time data. Furthermore, the obtained data can be analyzed easily using host computers. Integrating BIM with a GPS-based method would ease the flow of information between planned and as-built models and auto-track resources [33].

Table 4 depicts the studies conducted by GPS in the CPM field between 2007 and 2021. The work performed by Benham pointed out that a 3D assessment was conducted [33]. While to the best of the authors’ knowledge, no studies were conducted using 4D or 5D assessment. Regarding the number of 3D sensing technology used, the same study conducted by Benham was performed using GPS and image-based methods. While in terms of the scanning environment, the same study was performed outdoors [33].

Furthermore, some indicators of object recognition performance(i.e., recall, accuracy, and precision measures) were revealed. The study findings showed that the average overall recall, accuracy, and precision rate of four stages in a linear infrastructure project were 84.2, 80.9, and 85.4, respectively.

### 3.4. Image-Based Methods

An image-based method is common for providing onsite information by tracking progress and documenting it. Image-based systems are usually inexpensive and easy to use compared to other 3D sensing technologies. They can also generate the geometrical information of the 3D as-built model. Images, however, can be collected in different ways. On one hand, cameras collect images from the ground. Cameras can be monocular or stereo. Researchers pointed out taking several photos in and around the site to overcome the occlusions and the limited views [19,21,34,35,36,37,38,39,40,41,42,43,44,45].

On the other hand, UAVs collect images aerially; UAV mainly consists of high-resolution cameras and sensors. UAVs can fly over the site and cover the site and its surroundings [18,46,47,48,49,50,51,52,53].In addition, fewer researchers used both cameras and UAV as their data acquisition technology [54,55].

In all studies mentioned above, image-based methods use BIM to facilitate project status from site to office. The integration between those systems would also auto-monitor the project’s progress by comparing the BIM-based and the point-cloud models. 

Table 5 depicts the studies conducted by image-based methods in the CPM field between 2007 and 2021. Some studies were performed using 3D assessment [19,21,34,35,36,39,40,42,44,50,54,55]. At the same time, the remaining studies were performed using 4D assessment [18,37,38,41,43,46,47,48,49,51,52,53], while to the best of the authors’ knowledge, no studies were conducted using 5D assessment. Regarding the number of 3D sensing technology used, a few studies were performed using image-based and LS-based methods [39,46,47,55] However, a few studies were conducted indoors [18,35,50], while others were conducted outdoors.

**Figure 3:**
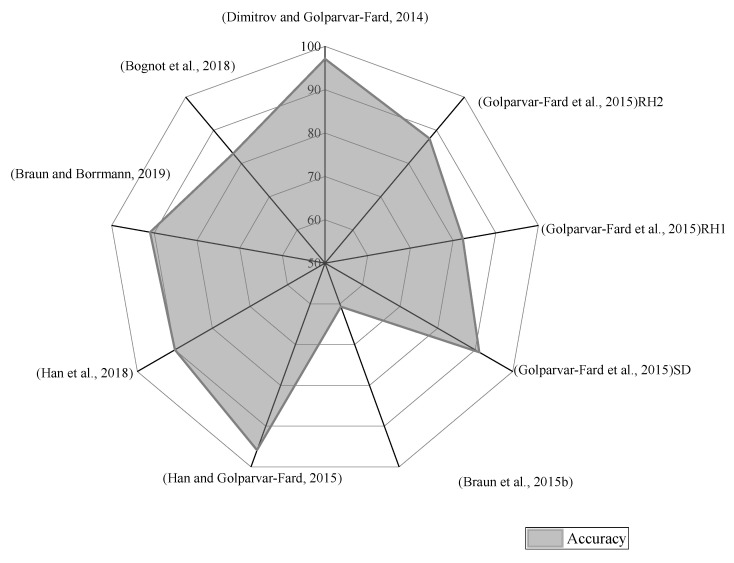
Accuracy (%) of studies [21,37,38,41,47,50,51] of image-processing in CPM.

Amongst these studies, a few studies revealed some indicators of object recognition performance (i.e., accuracy measure), as shown in Figure 3. The findings revealed that the result obtained in [21,41] had the highest accuracy with 97.1% and 95.9%, respectively. The remaining accuracy results fluctuated between 80%~90%, except the results obtained in [38], which had the lowest accuracy with 60.7%.

### 3.5. Laser Scanners

In recent years, the AEC industry has developed tremendously in using 3D scanning technologies in collecting the data of construction scenes, using only a few scans and images. A laser scanner is one of the best instruments to estimate construction project development using 3D point clouds to clarify the construction projects’ status [56]. However, as mentioned before, laser scanners were primarily used in surveying because of the large amount of data and the long computational time required. Laser scanners joined the monitoring and controlling stage as progress checkers due to the accuracy results of the 3D representation of the objects. The point clouds generated include two crucial information pieces. Firstly, each point cloud’s position information (x, y, and z). Secondly, digital cameras inside the laser scanner capture the color information (R, G, and B). Those are essential to detect buildings’ structure components [10,56]. Integrating laser scanners with BIM-based models would massively help to auto-detect the project’s progress. The integration between those systems would flow the information properly between the site and the office [10,23,30,39,46,47,55,56,57,58,59,60,61,62,63,64,65,66]. Table 6 depicts the studies conducted by laser scanners in the CPM field between 2007 and 2021. Some studies were performed using 3D assessment [23,30,39,47,55,57,58,59,60,65]. In comparison, fewer studies were conducted using 4D assessment [10,46,56,62,64,66]. While to the best of the authors’ knowledge, very few studies were performed using 5D assessment [63]. Shahi pointed out the usage of more than one 3D sensing technology where UWB-based systems and laser scanners were used together [30].

In contrast, a few studies were conducted using image-based methods and laser scanners [39,46,47,55]. However, some studies were conducted indoors regarding the scanning environment [58,59,60,62,65], while [23,61] was implemented indoors and outdoors. In contrast, the remaining studies were performed outdoors.

**Figure 4:**
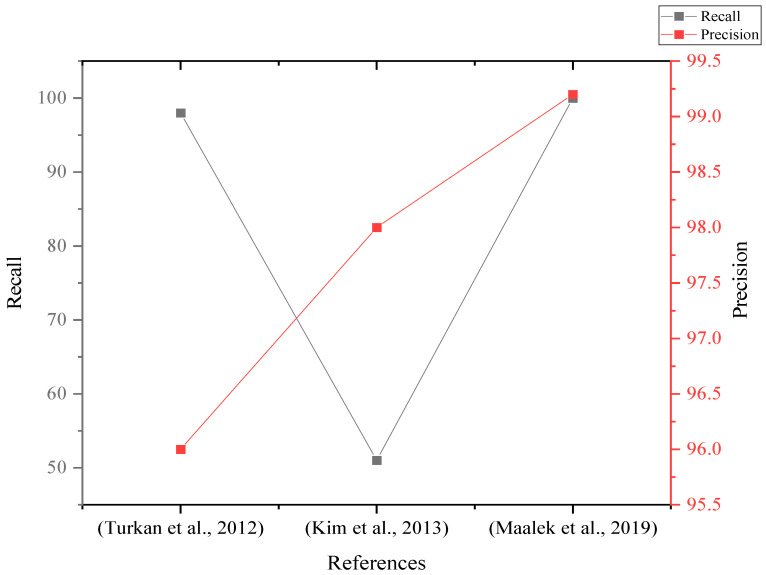
Recall and precision (%) of laser scanning studies [10,56,63] for construction progress monitoring.

Amongst these studies, a few studies used indicators of object recognition performance (i.e., recall, accuracy and precision) [10,56,63]. Those studies’ findings showed that the recall and the precision rates pointed out by Maleek [63] are higher than those pointed out by Kim and Turkan, as shown in Figure 4. However, the recall and precision results pointed out by Maleek were for columns only compared to a whole project addressed by Kim and Turkan.

## 4. Critical Analysis for Previous Studies

### 4.1. Summary of the Current State of the Art

Today, 3D sensing technologies can assist site engineers in automatically monitoring and controlling activities in construction projects. This article’s literature synthesis has been conducted for the past fifteen years. Forty-six studies have been collected. The literature showed that image-based and LS-based methods were the most utilized data acquisition technology, while GPS-based methods were the least used technology. In addition, studies that collect data using more than one data acquisition technology have increased in the last ten years, where the fusion between LS-based and image-based methods was the most common.

**Figure 5:**
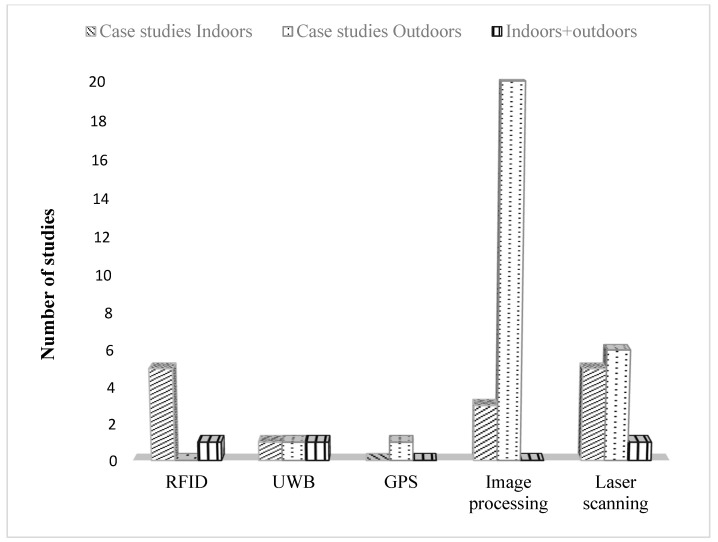
Scanning environment of 3D sensing technologies studies.

Furthermore, most studies preferred the outdoor environment, as shown in Figure 5. Figure 5 also revealed that most RFID, UWB, and GPS studies were conducted indoors. Therefore, researchers intend to use these technologies to track resources that are generally inside the site. On the contrary, most studies conducted by image-based methods were performed outdoors. Moreover, LS-based studies were performed in any scanning environment as the acquisition technology can detect components inside or outside the site. As a result, there is a correlation based on frequency between specific 3D sensing technologies and certain environments.

**Figure 6:**
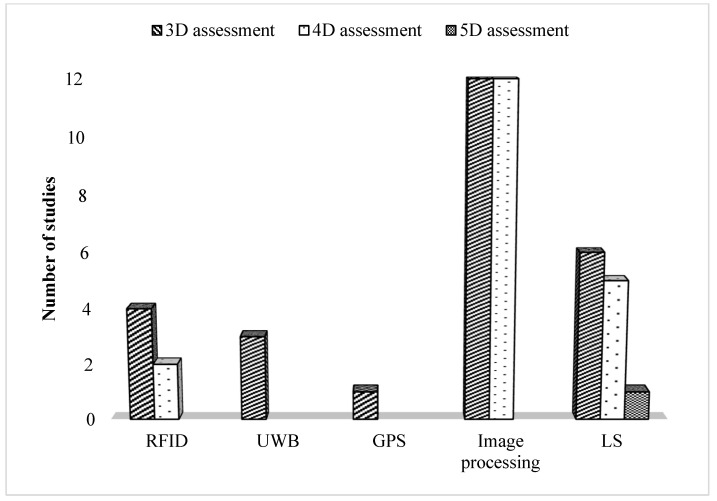
Level of assessment of 3D sensing technologies in CPM.

Moreover, this article revealed that almost all studies conducted with RFID, UWB, and GPS systems were performed using 3D assessment, as shown in Figure 6. Figure 6 also found that most studies conducted with image processing or laser scanning methods were performed using 4D assessment. However, only one study used laser scanning for using 5D assessment purposes. As a result, future work should focus mainly on using 5D assessment in the CPM domain integrated with BIM. There are other additional gaps in the collected studies. For example, integrating the BIM model and as-built model is easy to implement but sometimes unreliable. The reason behind that is when the distance between the as-planned location of the object is larger than the predefined spatial similarity criteria [63]. Another example is that most companies do not utilize 3D sensing technologies due to the high cost of technologies and equipment [10]. Software, tools, and algorithms are limited, and they need development to determine all the factors that could affect automated progress monitoring [17,18,19,20].

### 4.2. Statistical Analysis Using Meta-Analysis

#### 4.2.1. Meta-Regression Methods and Procedures

Meta-analysis is the evaluation of research findings from several empirical studies with the help of statistical tools [67]. On one hand, meta-analysis is a quantitative statistical tool to determine overall trends across studies [68]. On the other hand, the term meta-analysis is almost the entire systematic review process in a broader sense. Nevertheless, this article will apply meta-analysis as a statistical tool. The steps of the meta-analysis are then defined. The steps start with defining the research question, “determine the efficacy of object recognition indicators’ performance in the CPM”.

The steps proceed by conducting the eligibility criteria where the evaluation studies of interest contained at least two estimates of object recognition indicators’ performance (i.e., recall, accuracy, and precision).

As a result, 39 studies from 46 extracted studies were excluded using the eligibility criteria. Extracted data was then collected from the seven remaining studies for meta-analysis. Data were then included describing the evaluation study from which the main variable was derived. The main variable was expressed as the change in the efficacy of object recognition indicators’ performance coinciding with CPM applications integrated with BIM.

The steps moved forward to some concerns considered in this article, such as publication bias and heterogeneity. Publication bias tends to unpublish study findings if they are not statistically significant, unwanted, or difficult to explain [69]. In this research, retrieved data from evaluation studies were assessed for publication bias, where the “trim-and-fill” method was used numerically for the set of weighted effects [70]. The “trim-and-fill” method contains two steps. The first step is to trim the studies that cause a funnel plot asymmetry. As a result, the overall effect estimate produced by the remaining studies cannot be majorly impacted by the publication bias. The second step is to fill the missing studies in the funnel plot based on the bias-corrected overall estimate.

A heterogeneity test was also performed in this analysis, and then data were analyzed using a random effect model as the heterogeneity in these sets of effects was significant. Using a random effect model is justified. There are no systematic variations in the set of effects considered in fixed-effect models.

On the contrary, the random effect model recognizes the variation in effects as systematic. The difference in results between those two models is that the fixed effect model is unsatisfactorily conservative [71]. On the other hand, the outcomes in random effect models are conservative estimates of statistical significance to the detriment of the power to explain variance in effect size [72].

#### 4.2.2. Evaluation of Effect Size and Relative Weight

The change in object recognition indicators’ performances was interpreted as odd ratios. Thus, the basic effect was extracted from studies as follows:

(4) $$Effect\;size\;(ES)=\frac{\frac{TP}{FN}}{\frac{FP}{TN}}$$

where *ES* is the effect size, *TP* point clouds correctly sense the positive cases; *FN* point clouds incorrectly fail to reveal the presence of a point; *FP* wrongly shows a point cloud is present, and *TN* point clouds rightly sense the negative cases, for further explanation (see Section 3). Then, in the meta-analytic model, optimized weights are calculated in the following equation as these weights are the inverse variance [72]

(5) $$W_{Relative}=\frac{1}{{SE}^{2}}\;$$

where *W_Relative_* is the weight of an individual effect, and the *SE* is the standard error. The precision of effect size is the standard error index [73]. It was derived as follows:

(6) $${SE}^{2}=\frac{1}{TP}+\frac{1}{TN}+\frac{1}{FP}+\frac{1}{FN}\;$$

where *SE* is the standard error in studies, and *TP*, *TN*, *FP*, and *FN* are defined in (4).

Moving forward to calculate the overall true mean, the log odds method and the fixed-effect model was used as follows [73]:

(7) $${{ES}_{overall}=e}^{\frac{(\sum ln\;ES\;*W)}{\sum W}}\;$$

where *ES_overall_* is the weighted mean effect size, *e* is the exponential function; ln *ES*, the estimate of each effect size using natural logarithm; and *W* is the weight of each effect estimate (see Equations (4) and (5)). Equation (7) was then generated to estimate the overall estimates for the whole sample of individual effects.

Finally, the chi-squared distributed statistic *Q* was generated to evaluate the hypothesis that there is heterogeneity within study error as follows:

(8) $$Q=\sum w\;\times \;\left[ln\;ES-ln\;{ES}_{overall}\right]\;$$

*Q* can be considered a function of the weighted squared difference between the natural logarithm of study effect estimates, ln *ES* and the natural logarithm of the fixed overall effects. It was assumed that *Q* was significant (α = 0.01), heterogeneity was assumed, and a random effect model was chosen to calculate the weighted means.

#### 4.2.3. Deliverables

A meta-analysis of seven studies was conducted to estimate how the efficacy of object recognition indicators will be performed. Initially, the set of ESs is examined for heterogeneity using the chi-squared test. A large I^2^ signals that the percentage of total variation across studies is heterogeneous. The test results showed the heterogeneity in studies (I^2^ = 93.6%, *p* < 0.01). So, a random meta-analytic model was performed.

Table 7 shows the meta-analysis results to grasp the statistical data intuitively and visually. The first three columns show the reference of the study, the effect size, and the relative weight. Further, the table shows each study’s point estimate and 95% confidence intervals. Moreover, the last column shows the calculation of the overall *p*-value to determine if the extracted data from studies are statistically significant or not. A statistical significance exists if the *p*-value is lower than the significance level (α = 0.05). The results showed that the extracted data is statistically significant (*p*-value = 0.0003 < α = 0.05). The overall effect (in the last row) of object recognition indicators also showed an (11.84%) increase in performance in CPM applications (with 95% confidence between +3.12% and +45.22%).

**Figure 7:**
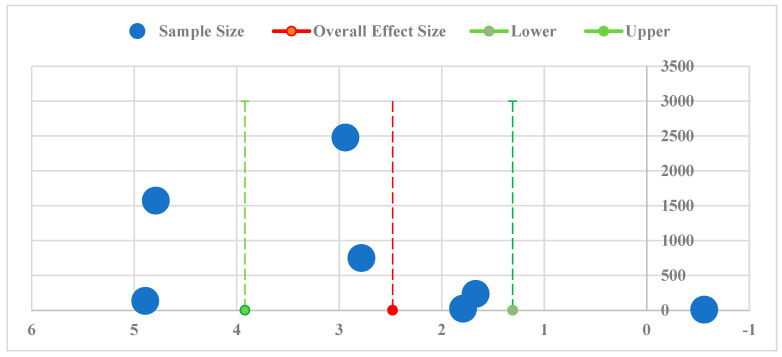
Funnel plot the natural logarithm of each effect size against its sample size.

Then, publication bias analysis was addressed to identify to which degree it influences the summary outcomes. Thus, the validity of the core findings was assessed. A funnel diagram is a common method to determine whether there is any publication bias. Figure 7 presents a funnel diagram drawn by plotting each effect size against its corresponding sample size to further analyze the publication bias in the whole sample. Figure 6 also shows that the data is consistent with the distribution of effects on either side of the overall effect size; however, the tails of the plot are not symmetrical, which is consistent with a hypothesis of publication bias.

Another reason is that most studies had a smaller sample size which occasionally indicates statistically insignificance; therefore, the trim and fill method was conducted to correct the effect size ES _corrected_ until the funnel was symmetric. The correction of effect size signifies that the object recognition indicators coincide with an 8.89% increase in performance in CPM applications where the confidence level is 95%, and the intervals are between +2.17% and 36.5%.

## 5. Conclusions

This research presents an interpretation of BIM in construction and the correlation between BIM and remote sensing in the CPM between 2007 and 2021. It also displays a critical analysis of previous studies using three main pillars: the scanning environment, level of assessment, and object recognition indicators’ performance. Synthesized literature was presented where forty-six studies were collected for the past fifteen years. Those studies used RFID, UWB, GPS, image processing, or laser scanning as data acquisition technology. Specified data was then extracted from these studies. Then, a critical analysis was performed using a meta-analysis model to evaluate the development of object recognition algorithms across the studies. This article also presented these studies’ strengths in different 3D sensing technologies flexibly used in CPM applications. The studies can also use different environment sets. This article found that most collected studies used either image processing or laser scanning methods for CPM applications. The article also showed a robust correlation between specific 3D sensing technologies and certain environment sets. It is found that there is a lack of studies performed using 5D assessment as well. Furthermore, the performance of object recognition indicators showed an increase of 8.89% across the studies where the estimated intervals were [2.17%,36.5%].

From these findings, it is recommended to focus on using the cost level of assessment in future progress monitoring applications. It is also preferred not to specify using one or two sensing technologies. The authors also suggest calculating at least one or two indicators of object recognition algorithms to determine the obstacles related to the integration between BIM and 3D sensing technologies and to propose solutions to better results of these indicators.

## Figures and Tables

**Table 1:** Confusion Matrix.

**Actual**	**Positive**	**Prediction**		**Negative**
		True	False	
		True Positive (TP)	False Negative (FP)	
		It happens when the presence of a point cloud is correctly predicted	It happens when a test fails to reveal the presence of a point cloud	
		False Positive (FP)	True Negative (TN)	
		It happens when a test incorrectly shows a point cloud is present	It happens when a test correctly predicts the absence of a point cloud	

**Table 2:** Studies of RFID integrated with BIM in CPM: 2007–2021.

	References	As-Planned vs. As-Built			Performance of Object(s) Recognition			Environment	Notes
		3D	4D	5D	Recall (%)	Accuracy (%)	Precision (%)		
1	[22]	**✓**	✓	⊠	N/A	N/A	N/A	Indoor	
2	[23]	✓	⊠	⊠	N/A	N/A	N/A	Indoor+Outdoor	It was performed using both RFID and laser scanner
3	[24]	✓	⊠	⊠	N/A	N/A	N/A	Indoor	
4	[25]	✓	⊠	⊠	N/A	N/A	N/A	Indoor	
5	[26]	✓	⊠	⊠	89.6	88.1	84.7	Indoor	
6	[27]	✓	✓	⊠	N/A	N/A	N/A	Indoor	

**Table 3:** Studies of UWB integrated with BIM in CPM: 2007–2021.

	References	As-Planned vs. As-Built			Performance of Object(s) Recognition			Environment	Notes
		3D	4D	5D	Recall (%)	Accuracy (%)	Precision (%)		
1	[29]	**✓**	⊠	⊠	N/A	N/A	N/A	Indoor+Outdoor	
2	[30]	✓	⊠	⊠	N/A	N/A	N/A	Outdoor	It was performed using both UWB and Laser scanner
3	[31]	✓	⊠	⊠	N/A	100 75	N/A	Indoor	The case study was conducted in two phases. One phase with LED indicator while the other phase without LED indicator

**Table 4:** Studies of GPS integrated with BIM in CPM: 2007–2021.

	References	As-Planned vs. As-Built			Performance of Object(s) Recognition			Environment	Notes
		3D	4D	5D	Recall (%)	Accuracy (%)	Precision (%)		
1	[33]	**✓**	⊠	⊠	84.8 73.1 81.1 97.8	80.3 72.1 76.9 94.2	89.6 72.7 83.7 95.7	Outdoor	It was conducted using both GPS and image-based method
					84.2	80.9	85.4		

**Table 5:** Studies of image-based methods integrated with BIM CPM: 2007–2021.

	References	Equipment		As-Planned vs. As-Built			Performance of Object(s) Recognition			Environment	Notes
		UAV	Camera	3D	4D	5D	Recall (%)	Accuracy (%)	Precision (%)		
1	[34]		✓	✓	⊠	⊠	N/A	N/A	N/A	Outdoor	
2	[35]		✓	✓	⊠	⊠	N/A	N/A	N/A	Indoor	
3	[21]		✓	✓	⊠	⊠	N/A	97.1	N/A	Outdoor	
4	[36]		✓	✓	⊠	⊠	N/A	N/A	N/A	Outdoor	
5	[37]		✓	✓	✓	⊠	N/A	87.5 82.89 91.05	N/A	Outdoor	Golparvar-Ford performed three case studies. Code names were given to these case studies which are RH1, RH2, and SD
6	[19]		✓	✓	⊠	⊠	N/A	N/A	N/A	Outdoor	
7	[46]	✓		✓	✓	⊠	N/A	N/A	N/A	Outdoor	It was conducted using image-based methods and laser scanning
8	[38]		✓	✓	✓	⊠	N/A	60.7	N/A	Outdoor	
9	[40]		✓	✓	⊠	⊠	N/A	N/A	N/A	Outdoor	
10	[39]		✓	✓	⊠	⊠	N/A	N/A	N/A	Outdoor	It was conducted using both image-based method and laser scanning
11	[41]		✓	✓	✓	⊠	N/A	95.9	N/A	Outdoor	
12	[54]	✓	✓	✓	⊠	⊠	N/A N/A	N/A N/A	N/A N/A	Outdoor	
13	[18]	✓		✓	✓	⊠	N/A	N/A	N/A	Indoor	
14	[47]	✓		✓	✓	⊠	N/A	90	N/A	Outdoor	It was conducted using Image-based and laser scanning methods
15	[48]	✓		✓	✓	⊠	N/A	91	N/A	Outdoor	
16	[49]	✓		✓	✓	⊠	N/A	N/A	N/A	Outdoor	
17	[50]	✓		✓	⊠	⊠	N/A	N/A	N/A	Indoor	
18	[51]	✓		✓	✓	⊠	N/A	82~84	50~72	Outdoor	
19	[55]	✓	✓	✓	⊠	⊠	N/A	N/A	N/A	Outdoor	It was conducted using both image-based method and laser scanning
20	[42]		✓	✓	⊠	⊠	79.5 79.1	N/A N/A	93.9 90.7	Outdoor	There were two case studies, Project 1 and project 2
21	[43]		✓	✓	✓	⊠	N/A	N/A	N/A	Outdoor	
22	[52]	✓		✓	✓	⊠	N/A	N/A	N/A	Outdoor	
23	[53]	✓		✓	✓	⊠	N/A	N/A	N/A	Outdoor	
24	[44]		✓	✓	⊠	⊠	N/A	N/A	N/A	Outdoor	

**Table 6:** Studies of laser scanning integration with BIM in CPM: 2007–2021.

	References	As-Planned vs. As-Built			Performance of Object(s) Recognition			Environment	Notes
		3D	4D	5D	Recall (%)	Accuracy (%)	Precision (%)		
1	[23]	✓	⊠	⊠	N/A	N/A	N/A	Indoor+Outdoor	Mentioned before, in Table 2
2	[10]	✓	✓	⊠	98	N/A	96	Outdoor	
3	[57]	✓	⊠	⊠	N/A	N/A	N/A	Outdoor	
4	[56]	✓	✓	⊠	51	N/A	98	Outdoor	
5	[58]	✓	⊠	⊠	N/A	N/A	N/A	Indoor	
6	[59]	✓	⊠	⊠	N/A	N/A	N/A	Indoor	
7	[60]	✓	⊠	⊠	N/A	N/A	N/A	Indoor	
8	[30]	✓	⊠	⊠	N/A	N/A	N/A	Outdoor	Mentioned before, in Table 3
9	[61]	✓	⊠	⊠	N/A	N/A	N/A	Outdoor +Indoor	
10	[46]	✓	✓	⊠	N/A	N/A	N/A	Outdoor	Mentioned before, in Table 5
11	[39]	✓	⊠	⊠	N/A	N/A	N/A	Outdoor	Mentioned before, in Table 5
12	[62]	✓	✓	⊠	N/A	N/A	N/A	Indoor	
13	[47]	✓	⊠	⊠	N/A	68	N/A	Outdoor	Mentioned before, in Table 5
14	[63]	✓	✓	✓	100	99.3	99.2	Outdoor	The set of results is only for columns.
15	[64]	✓	✓	⊠	N/A	N/A	N/A	Outdoor	
16	[55]	✓	⊠	⊠	N/A	N/A	N/A	Outdoor	Mentioned before in Table 5
17	[65]	✓	⊠	⊠	N/A	N/A	N/A	Indoor	
18	[66]	✓	✓	⊠	N/A	N/A	N/A	Outdoor	

**Table 7:** Results from Random effect meta-analysis model.

	Study	Effect Size (ES.)		% Changes in Object Recognition Indicators’ Performance			*p*-Value
			Relative Weight	Lower 95%	Estimate	Upper 95%	
1	[26]	−0.56	0.161	+0.27	+0.6	+1.17	
2	[33]	2.78	0.159	+7.30	+16	+36	
3	[38]	1.79	0.135	+1.2	+6	+30.63	
4	[49]	2.93	0.168	+15	+19	+23.7	
5	[57]	4.79	0.129	+19.6	+120	+735	
6	[56]	1.66	0.138	+1	+5.3	+24.75	
7	[64]	4.89	0.109	+7.29	+133	+1480	
		**2.48**		**+3.102**	**+11.84**	**+45.22**	**0.0003**
